# Scientists disrupt less but more persistently with career aging

**DOI:** 10.1073/pnas.2626763123

**Published:** 2026-10-19

**Authors:** Nan Deng, Liying Yang, Shlomo Havlin, An Zeng

**Affiliations:** ^a^ National Science Library, Chinese Academy of Sciences, Beijing 100190, China; ^b^ Department of Information Resources Management, School of Economics and Management, University of Chinese Academy of Sciences, Beijing 100190, China; ^c^ Department of Physics, Bar-Ilan University, Ramat-Gan 5290002, Israel; ^d^ School of Systems Science, Beijing Normal University, Beijing 100875, China

**Keywords:** science of science, persistent disruption, scientific career, aging effect, scientific impact

## Abstract

Disruption describes the extent to which a scientific work overturns the prior knowledge on which it builds and leads to a new line of research. A scientist’s tendency to produce disruptive work has been found recently to decline over the career. However, the disruption index captures only how strongly a paper overturns prior work and ignores whether that disruption is later accepted or overturned by subsequent research. The persistent disruption (PD) framework adds this second perspective, identifying work that disrupts prior research and is then consolidated rather than displaced by later work. We examine how PD evolves over the career and find the opposite pattern: The probability of producing a persistent disruptive paper increases with career age. Senior scientists disrupt prior work less often compared to young, but once they do, the disruption tends to persist rather than being quickly superseded. Our results suggest that this reversal feature is due to the experience and we corroborate it across several dimensions of recognition. We also show that, at a fixed level of disruptiveness, the more senior the scientist is, it attracts more citations, social-media attention, and research grants. Nobel laureates also show delayed persistent disruptions. They exhibit high disruptive ability early in their careers, yet their prize-winning work appears years later. This lag further indicates that experience is needed to turn disruptive ability into the field’s most celebrated contributions.

Disruptive research—discoveries that overturn prior foundations rather than incrementally building upon them—drives scientific innovation and progress (1, 2), and understanding it has become a central concern in the research field science of science. A paper is considered disruptive when later papers cite it without also citing the earlier studies on which it builds, indicating that it has displaced rather than consolidated its predecessors. A large literature in science of science has sought to understand scientific creativity and innovation across researchers’ careers. Scientific impact can emerge at any career stage (3) and often clusters within temporally localized hot streaks (4), during which disruption is also elevated (5). Nobel-prize-winning work, however, has shifted toward later career ages (6), while scientists increasingly change research topics as their careers advance (7).

Recent work reports that disruptive output declines as scientists age (8) and is less common among more productive scientists (9). Yet the conventional disruption index has a blind spot: It measures only how strongly a paper overturns prior work and not whether that disruption itself endures. A paper may disrupt earlier research only to be sooner or later disrupted in turn. The persistent disruption (PD) framework and measure close this gap by adding a second dimension that captures whether a disruptive paper is later consolidated rather than displaced by the work that cites it. Indeed, persistently disruptive papers are more likely to become lasting and award-winning contributions (10).

However, how persistent disruption evolves over the scientific career of a scientist remains unknown. Here, we show that the aging effect on persistent disruption of a scientist runs opposite to its effect on disruption: Although scientists disrupt less as they age, the disruptions they do produce are increasingly likely to persist, and we trace this reversal to the accumulation of experience.

## Results

### The Aging Effect on Persistent Disruption.

**Fig. 1.**
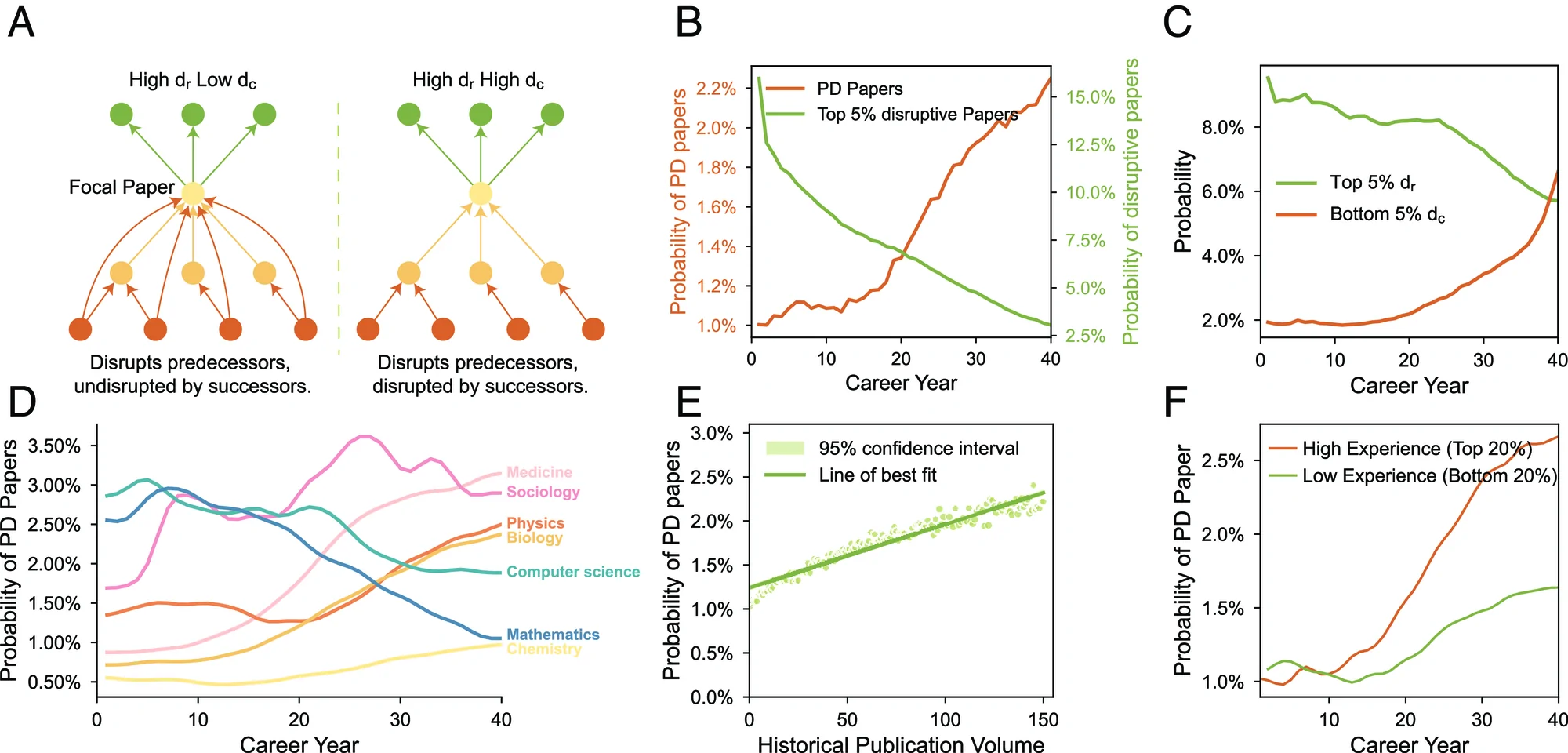
The aging effect on persistent disruption. (*A*) Persistent disruption (PD) combines reference disruption *d*_*r*_ and citation disruption *d*_*c*_. (*B*) With career year, the probability of publishing a top-5% disruptive paper declines while that of a PD paper rises. (*C*) Decomposed by dimension: The probability of reaching the top 5% in *d*_*r*_ decreases, whereas that of reaching the bottom 5% in *d*_*c*_ increases. The patterns in (*B* and *C*) are robust to calendar-time adjustment, consistent across entry cohorts, career-length samples, and PD thresholds (*SI Appendix*). (*D*) PD probability by field across career year. (*E*) Papers grouped by their authors’ cumulative prior publications (*x*) versus mean PD probability (*y*). (*F*) Within each career year, PD probability for a high-experience cohort (top 20% by output) versus a low-experience cohort (bottom 20% by output).

Persistent disruption (PD) is based on a two-dimensional framework (10). Its two dimensions are reference disruption (*d*_*r*_), measuring how much a focal paper disrupts its references, and citation disruption (*d*_*c*_), measuring how much the paper is subsequently disrupted by its citing papers (see *SI Appendix* for details). We classify a paper as PD paper when *d*_*r*_ lies in the top 5% and *d*_*c*_ in the bottom 5%. Such a paper displaces prior work without being rapidly displaced in turn (Fig. 1*A*). PD thus filters out nonreal disruption, work that overturns previous research only to be quickly overturned in turn.

Using this PD framework, we find that career aging acts on disruption and persistent disruption in opposite directions. As career year increases, the probability of publishing a top 5% disruptive paper declines, consistent with the documented erosion of disruptive output over a career (8). By contrast, the probability of publishing a PD paper increases with career year, from roughly 1% at the start of a career to about 2.2% by career year 40 (Fig. 1*B*). Decomposing PD into its two dimensions clarifies this contrast: The probability of reaching the top 5% in *d*_*r*_ decreases with career year, indicating that scientists become less likely to produce work that strongly displaces prior knowledge, whereas the probability of reaching the bottom 5% in *d*_*c*_ increases (Fig. 1*C*), indicating that their work becomes less likely to be disrupted in turn. Taken together, although scientists disrupt less often as they age, their disruptive contributions become increasingly likely to endure and be built upon.

This aging effect is not uniform across fields (Fig. 1*D*). In most disciplines, including medicine, sociology, physics, biology, and chemistry, PD probability keeps rising in later career stages. However, computer science and mathematics are the exceptions, where PD probability also decreases over the career. One possible explanation is that breakthroughs in these fields rely more heavily on individual insight and less on accumulated experience, resources, or reputation.

To understand the overall rise of PD with career age, we examine whether it is associated with cumulative publication experience. An author’s cumulative publication volume, used here as a proxy for cumulative publication experience, is indeed positively associated with the probability of producing a PD paper (Fig. 1*E*). Furthermore, holding career stage fixed, the effect persists: Even within the same career year, a high-experience cohort has a substantially higher probability of producing PD papers than a low-experience cohort, and this gap widens with career year (Fig. 1*F*, see *SI Appendix* for details). Cumulative publication experience, rather than career age alone, thus helps account for the rise of persistent disruption.

### Support from Citation, Media, and Funding Impact, and the Nobel Prize.

**Fig. 2.**
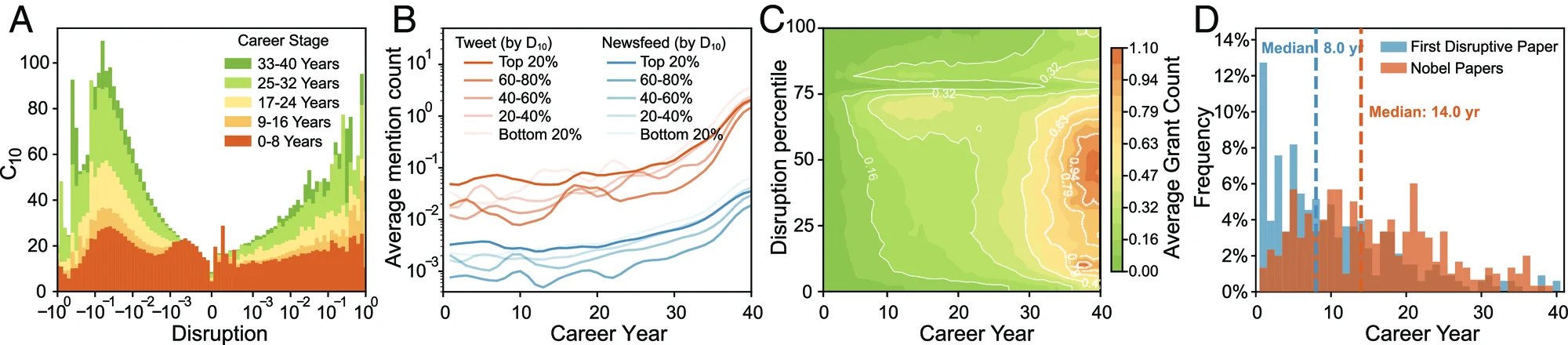
Disruptions by senior scientists are more readily accepted by the community. (*A*) Controlling for *D*_10_, more senior career-age groups receive more citations *C*_10_. (*B*) Within each *D*_10_ group, more senior scientists receive higher tweet and newsfeed counts. (*C*) Within each *D*_10_ group, more senior scientists receive more NSF and NIH grants per paper. (*D*) For Nobel laureates, the career-year distribution of their first top 5% disruptive paper (blue) peaks about six years earlier than that of their prize-winning paper (red).

Having established that = cumulative publication experience is associated with publishing more PD papers, although disruption (*D*) declines with age (8), we hypothesize that, once a senior scientist does produce a disruptive paper, that disruption is more readily recognized by the community—which would turn it into a PD paper, a high-quality, lasting contribution. To isolate this effect from age’s opposing influence on disruption, we control for *D*_10_ and examine whether seniority still promotes recognition. Controlling for *D*_10_, papers produced by more senior scientists receive more citations (*C*_10_) (Fig. 2*A*), consistent with reputation driving citation impact in academic careers (11). The same holds for social impact: Within each *D*_10_ group, more senior scientists receive higher tweet and newsfeed counts (Fig. 2*B*), and for institutional endorsement, measured by NSF and NIH grants per paper (Fig. 2*C*). Together, these results suggest that, at comparable levels of disruption, work by more senior scientists is more likely to receive attention and support that may help its influence endure. These advantages in reputation and resources may also increase the likelihood that disruptive work becomes persistently disruptive (see *SI Appendix* for details).

Producing Nobel-level work also requires the accumulation of experience. Laureates’ prize-winning papers are known to exhibit exceptionally high persistent disruption (10), yet the career-year distribution of their first top 5% disruptive paper peaks about six years earlier than that of their prize-winning paper (Fig. 2*D*). That laureates already disrupt strongly early in their careers but produce their prize-winning work years later suggests that early disruption may require accumulated experience to mature into persistent disruption. (see *SI Appendix* for details).

## Discussion

Our study reveals an interesting aging effect that complicates the prevailing narrative of declining innovation over a scientific career (8). Scientists may become less likely to produce disruptive work as they age, yet the disruptive work they produce is more likely to persist. Our central message is that cumulative publication experience makes scientists’ disruptive papers more persistent rather than publishing more disruptive papers. These findings should be interpreted with several qualifications. First, disruption-based metrics have known limitations, and cumulative output and career age capture only part of what scientists accumulate over a career. Second, citation dynamics vary across disciplines, and a fixed 10-y citation window may not be adequate for all disciplines studied. Third, the relationships we report are associational rather than causal. With these qualifications, evaluation and funding systems focused only on immediately visible disruption may overlook the contribution of accumulated experience to the durability of scientists’ disruptive ideas.

## Materials and Methods

### Datasets.

We draw on SciSciNet, a large-scale open bibliographic data lake for science of science (12). From it we select 206,130 scientists whose careers span more than 40 y, comprising 11,203,206 papers, as our analysis sample. We measure citation impact by *C*_10_ (citations within a 10-y window) and disruptiveness by *D*_10_ (disruption within a 10-y window), and use tweet and newsfeed counts as a proxy for social impact and the number of NSF and NIH grants per paper as a proxy for funding recognition. For the Nobel analysis, we use the prize-winning papers of 362 laureates whose careers span over 40 y. See *SI Appendix* for the detailed data descriptions.

### Disruption and Persistent Disruption.

Conventional disruption (*D*_10_) quantifies how much subsequent papers within 10 y cite a focal paper while bypassing its references. Persistent disruption additionally asks whether that disruptive position endures. A persistently disruptive paper disrupts the prior work on which it builds but is not itself *displaced* by later research. Operationally, this corresponds to two indices: high reference disruption (*d*_*r*_) and low citation disruption (*d*_*c*_); detailed definitions are provided in *SI Appendix*.

## Supplementary Material

Appendix 01 (PDF)

## Data Availability

The data and code can be accessed in Zenodo: https://doi.org/10.5281/zenodo.20527134 (13).
