# Supplementary material for: Scientists disrupt less but more persistently with career aging
Source: Proc Natl Acad Sci U S A. 2026 Oct 19;123(43):e2626763123. doi: 10.1073/pnas.2626763123 (PMC13715735; doi:10.1073/pnas.2626763123)
Supplement: Supplementary file 1 [file pnas.2626763123.sapp.pdf]

Supporting Information for

Scientists disrupt less but more persistently with career aging

Nan Deng, Liying Yang, Shlomo Havlin and An Zeng

Shlomo Havlin
E-mail: havlins@gmail.com

An Zeng
E-mail: anzeng@bnu.edu.cn

This PDF file includes:

Supporting text
SI References

Supporting Information Text

S1. Data and preprocessing

Our analysis is based on SciSciNet, a large-scale open data lake for the science of science built on the Microsoft Academic Graph (1). It covers over 134 million scientific publications, together with their citation links, disambiguated authors, and external linkages to funding and social-media records.

Our analysis draws on the author–paper relationships in SciSciNet, from which we additionally compute a set of career-related variables, including each author's first and last publication years (`Career_Start` and `Career_End`) and a paper's *career year* (`Career_Year`), defined as the number of years since the author's first publication, with a paper published in the author's first year assigned career year 1. Full data descriptions are provided in the accompanying data repository (2).

To capture the full arc of a career without truncation, we restrict our analysis to authors whose careers span more than 40 years, yielding 206,130 scientists and 11,203,206 distinct papers. For the Nobel analysis, we use the prize-winning papers of 362 laureates whose careers span more than 40 years.

For each paper in this sample, we retain from SciSciNet its number of tweets and newsfeeds mentioning it (`Tweet_Count` and `Newsfeed_Count`), and the number of acknowledged NSF and NIH grants (`NSF_Count` and `NIH_Count`). In addition, we compute from each paper's citation records the 10-year disruption index D_{10}, the 10-year citation count C_{10}, and the two dimensions of persistent disruption, namely the reference disruption d_r and the citation disruption d_c. We classify a paper as highly disruptive if its D_{10} falls in the top 5% of the sample.

S2. Measuring disruption

The conventional disruption index D_{10} (3, 4) is calculated using citations accumulated within ten years after publication.

$$D_{10} = \frac{n_i - n_j}{n_i + n_j + n_k},$$

where n_i is the number of papers cite focal paper but none of its references, n_j is the number citing both focal paper and its references, and n_k is the number of subsequent papers that cite references only. Papers without a complete 10-year citation window are excluded according to the actual procedure.

S3. Measuring persistent disruption

We adopt the persistent disruption framework (5). We first compute disruption at the level of individual citation links. For a focal paper i and a reference j it cites, the link-level disruption is

$$d_{ij} = \frac{n_d - n_c}{n_d + n_c + n_r}, \qquad [1]$$

where n_d is the number of papers that cite i but not j, n_c is the number that cite both i and j, and n_r is the number that cite j but not i. A value close to 1 indicates that i disrupts reference j and redirects subsequent attention to itself, whereas a value close to -1 indicates that i consolidates the paradigm established by j.

By aggregating this link-level indicator over a paper's references and over its subsequent citing papers, we obtain two dimensions. Averaging d_{ij} over all references that paper i cites gives the *reference disruption* $d_r = \frac{1}{|R_i|}\sum_{j \in R_i} d_{ij}$, which measures how strongly i disrupts the prior work it builds on. By the same construction, paper i is itself a reference for the papers that later cite it; averaging the d_{ij} over all citing papers gives the *citation disruption* $d_c = \frac{1}{|C_i|}\sum_{i \in C_i} d_{ij}$, which measures how strongly i is disrupted by the work that follows it. A high d_r therefore reflects a strong disruption to the past, while a low d_c reflects that the disruption persists rather than being disrupted in turn.

A persistently disruptive (PD) paper is one that both highly disrupts prior work and is not itself displaced by later work. We set the threshold for a PD paper as being in the top 5% of d_r and simultaneously in the bottom 5% of d_c. PD thus rejects spurious disruption, in which a paper disrupts prior work only to be disrupted later, filtering only disruptions that are consolidated by the subsequent literature.

S4. Measuring cumulative publication experience

We use an author's cumulative publication count as a proxy for accumulated publication experience. For a given paper, this is the number of papers the author had published before the focal paper, with all co-authored papers counted.

For the cohort analysis (main-text Fig. 1f), within each career year we split papers into a *high-experience cohort* and a *low-experience cohort*. The high-experience cohort consists of papers whose authors' cumulative output exceeds that of 80% of their peers in the same career year (i.e., the top 20% by output); the bottom 20% form the low-experience cohort.

S5. Measuring community recognition

To assess how strongly a scientist's work is recognized by the scientific community, we draw on measures along several dimensions. The most basic is *citation*: we use the 10-year citation count C_{10} to capture recognition within the scientific literature. Beyond citations, we use the number of tweets and newsfeeds mentioning a paper (`Tweet_Count` and `Newsfeed_Count`) as a measure of

its social attention and the number of acknowledged NSF and NIH grants (`NSF_Count`, `NIH_Count`) as a measure of institutional recognition and support. Finally, we treat the Nobel Prize as an elite and highly selective form of scientific recognition. Together, these measures span recognition at different levels, from individual papers to the most celebrated achievements.

For the citation, media, and funding analyses (Fig. 2a–c), disruption acts as a confounder. We therefore control for D_{10} and compare, at the same level of disruption, the recognition received by authors at different career stages. If senior authors attract more citations, social attention, and funding even when their work is equally disruptive, this indicates that recognition accrues with experience beyond what disruptiveness alone explains. This provides support from the dimension of community recognition, for the pattern documented in Fig. 1, namely that experience makes a scientist's disruptions more likely to be accepted and thus to become persistent.

For the Nobel analysis (Fig. 2d), we define a laureate's *first disruptive paper* as the first paper in their career that falls in the top 5% of D_{10}. The career-year distribution of these first disruptive papers indicates when laureates begin to display disruptive ability. By overlaying the career-year distribution of their prize-winning papers, we compare the onset of disruptive ability with the timing of their most celebrated work. The two distributions reveal that, even when laureates display disruptive ability early, their prize-winning work tends to appear only some years later, indicating that turning disruptive ability into the community's highest recognition may require the further accumulation of experience.

S6. Robustness analyses

Calendar-time, cohort, and career-length robustness. Because career age is correlated with calendar time, we tested whether publication-year trends or cohort differences accounted for the increase in PD. We adjusted PD using the annual paper-level baseline and examined career-age trends within the 1980–1999 entry cohorts. The association between career age and PD remained positive after publication-year adjustment and was present across most cohorts.

The main analysis includes scientists with careers longer than 40 years. To assess sensitivity to this sample definition, we repeated the analysis using career-length thresholds of 10, 20, and 30 years, as well as an unrestricted sample. Across these samples, PD probability increased with career age, whereas the probability of a highly disruptive paper declined.

Alternative thresholds for persistent disruption. We examined the robustness of the main results across alternative PD thresholds. We repeated the analysis using 1%, 10%, 15%, and 20% thresholds for high d_r and low d_c. The same career-age pattern was observed: PD probability increased with career age while the probability of highly disruptive papers declined. The separate patterns of d_r and d_c were also preserved across these alternative thresholds.

We further constructed continuous measures by varying the paired d_r and d_c thresholds from 1% to 20%. For each paper, the continuous PD score records how often it satisfies the corresponding PD condition across these thresholds, such that papers satisfying more stringent conditions receive higher scores. Using these continuous measures, persistent disruption increased with career age, whereas disruption declined.

Reputation, resources and persistent disruption. We further examined whether the citation, media, and grant advantages associated with seniority may help disruptive work become persistently disruptive. Within each career year, papers were divided into 50 percentile bins according to their disruption level. High- and low-advantage papers were then compared within the same career year and similar disruption levels, and the two groups were standardized to the same disruption distribution.

At comparable career stages and disruption levels, papers with greater citation impact, media attention, and grant support were more likely to become persistently disruptive.

Randomized null model for Nobel lag. Because the first highly disruptive paper is mechanically more likely to occur early in a career, we constructed a randomized null model for the timing analysis of Nobel laureates. For each laureate, we retained the observed publication sequence and Nobel-paper timing, but randomly permuted the highly disruptive-paper labels across the career. We then compared the observed lag between the first highly disruptive paper and the Nobel-recognized paper with the randomized distribution.

The observed mean lag was 7.02 years, compared with 4.18 years under the null model (one-sided Monte Carlo test, $P = 0.0001$, 10,000 randomizations). The same pattern was obtained when timing was measured by publication order rather than career year. Thus, the observed separation between early disruption and later Nobel-recognized work is larger than expected from the mechanical ordering of a first disruptive event alone. This suggests that, beyond this mechanical effect, disruptive ability may require time to mature into prize-winning work.

References

1. Z Lin, Y Yin, L Liu, D Wang, SciSciNet: A large-scale open data lake for the science of science research. *Sci. Data* **10**, 315 (2023).
2. N Deng, Data and code for: Scientists disrupt less but more persistently with career aging (2026) Zenodo. https://doi.org/10.5281/zenodo.20527134. Deposited 22 June 2026.
3. RJ Funk, J Owen-Smith, A dynamic network measure of technological change. *Manag. Sci.* **63**, 791–817 (2017).
4. L Wu, D Wang, JA Evans, Large teams develop and small teams disrupt science and technology. *Nature* **566**, 378–382 (2019).
5. N Deng, X Gu, Y Fan, S Havlin, A Zeng, The critical role of persistent disruption in advancing science. *Nat. Comput. Sci.* **5**, 492–501 (2025).
